# The Effect of Cabergoline on Clinical and Laboratory Findings in Active Rheumatoid Arthritis

**Published:** 2011-10-01

**Authors:** M Mobini, Z Kashi, A R Mohammad Pour, E Adibi

**Affiliations:** 1 Department of Internal Medicine, Imam Khomeini Hospital, Mazandaran University of Medical Sciences, Sari, Iran

**Keywords:** Rheumatoid arthritis, Prolactin, Cabergoline, Dopamine agonist

Dear Editor,

Rheumatoid arthritis (RA) is a chronic systemic inflammatory disorder affecting about 1% of the world population.[1] In recent years, biologic agents were introduced for many aspects of medicine including RA, but their cost and potential side effects prohibited its widespread use.

Dopamine agonists such as bromocriptine and cabergoline decreased the prolactin synthesis and secretion by binding of the drug to cell-surface dopamine receptors.[2]

Prolactin is secreted not only by anterior pituitary gland, but also by immune cells and it was shown that it can stimulate the immune cells by binding to prolactin receptors.[3] Prolactin receptors were exclusively expressed on fibroblast like synovial cells and lymphocytes infiltrating into the synovium in patients with RA.4 It has demonstrated that PRL can enhance RA synovial cell proliferation. There are four open trials about bromocriptine[5][6][7][8] and one case report about cabergoline[9] in RA treatment. Cabergoline is an ergot dopamine agonist that is administered once or twice a week and has much less tendency to cause nausea than bromocriptine.[10]

This study is a pilot randomized double blind clinical trial carried out from September 2009 to May 2010, on 10 patients with active RA who had been referred to Rheumatology Clinic in Sari, Iran. RA was defined according to ACR in 1987 and criteria for disease activity included existence of 4 swollen joints plus 2 of the followings criteria (i) Existence of 6 tender joints, (ii) Morning stiffness more than 30 minutes and (iii) Erythrocyte sedimentation rate (ESR) more than 28 mm/h.[11][12]

The patients suffered from active RA despite receiving prednisolone and DMARDs for at least 3-6 months. The study was approved by Ethics Committee of Mazandaran University of Medical Sciences and was recorded in IRCT (IRCT code: IRCT138802061828N2). All patients signed informed consent. Patients with psychosis, pregnancy or lactation were excluded from the study. Patients continued their drugs with the same dose and kind of DMARD.

Patients were randomly divided into two groups to receive 1 mg/week of cabergoline (Pharmacia and Upjohn SPA, Italy) or placebo (Sari, Iran, Pharmacy Faculty). The two groups were similar in terms of disease duration and activity and main antirheumatoid therapy. In first step, patients took cabergoline or placebo for 3 months and after 1 month, they used another drug for another 3 months period. Changes in disease activity in the beginning of the study and at 3rd, 4th and 7th month of treatment and possible side effects were recorded.

Statistical analysis was done by t-test for quantitative variables; pair t test for comparison before and after interventions, and Wilcoxon signed test and Friedman for non-parametric methods. Non parametric statistical analysis was done by Wilcoxon signed and Friedman Exact test for comparison before and after interventions.

Ten female patients with active RA entered this study. The study was followed for 9 patients. One patient in first group was excluded because of complain of vertigo and vomiting. The mean age of patients was 55.6±9.5 years, mean of disease duration was 12.1±6.0 (years), morning stiffness of 30.5±41.2 minutes, tender and swollen joint count of 7.4± 2.8 and 5.1±1.8, and patient assessment of pain and global assessment of disease activity were 6.4±3.0 and 5.5±2.9 according to visual analogue scale(VAS). PRL level was 9.4±7.0 (ng/ml) and mean for ESR was 35.0±14.4 (mm/h). After intervention by cabergoline, prolactin level decreased from 10.6±4.3 to 6.4±5.8 (ng/ml) (p=0.188) and by placebo it increased from 9.9±10.7 to 15.0±8.4 (ng/ml) (p=0.375). We compared changes in diseases activity by cabergoline and placebo as shown in Table 1.

**Table 1:** Comparison of changes in disease activity by cabergoline and placebo.

**Variable**	**Beforecabergoline**	**Aftercabergoline**	**P value**	**Beforeplacebo**	**Afterplacebo**	**P value**
Morning stiffness (min)	17.8±28.5	25.6±45.7	1.000	18.3±29.5	30.0±78.8	1.000
Tender joint count	6.7±3.6	2.7±2.3	0.011	3. 9±4.4	3.0±2.9	0.594
Swollen joint count	4.1±2.6	2.0±1.6	0.031	2.7±3.4	1.8±2.8	0.344
Patient assessment of	5. 6±3.5	3.2±2.4	0.047	4.9±3.4	4.1±3.6	0.438
pain (vas)						
Patient global assessment	4.67±3.2	2.6±1.8	0.516	4.2±3.3	3.8±3.7	0.625
of disease activity (vas)						
ESR (mm/h)	41.0±18.6	29.8±16.1	0.156	24.7±18.3	30.7±23.7	0.438

In this study, improvement of tender and swollen joint count, patient assessment of pain and patient global assessment of disease activity were significant when patients were treated by cabergoline. Prolactin is secreted not only by anterior pituitary gland, but also by immune cells that may have small effect on total serum prolactin level and a significant effect on immunomedulatory system, so cabergoline suppressed both kinds of prolactin. The improvement in RA activity may be due to a significant suppression in secretion of prolactin by immune cells without a significant change in prolactin level. Thus, we noticed significant improvement in patients receiving cabergoline without a large decrease in prolactin level. Dougados et al. did not find any difference by bromocriptine in clinical and laboratory measures of the disease activity in 6 RA patients.[5] Another study by Marguerie et al. in 30 patients with active RA showed some clinical improvement by bromocriptine in comparison to penicillamine.[6] Mader and Figueroa demonstrated clinical improvement by bromocriptine too.[7][8]

Erb and coworkers reported a patient with severe uncontrolled RA that improved rapidly after treatment for coincidental hyperprolactinemia,[9] and Eijsbouts et al. tried on quinagolide for 6 months and despite suppression of PRL level, there was not any improvement in clinical or laboratory findings.[13] This study is the first, clinical trial about cabergoline in RA. It was a small and pilot study and we suggest future studies with more samples and with different dosages and intervals of prolactin inhibitors especially cabergoline. Two of the patients did not have a good tolerance with 1 mg/week of cabergoline, thus, we suggested administration of a lower dosage and a 2 times/week. Cabergoline is long acting and its usage and cost is acceptable and it has little complications. These factors are reasonable to study more about cabergoline in RA and other rheumatologic disorders that formerly have shown improvement by dopamine agonists.
